# Synergism of amlodipine and telmisartan or candesartan on blood pressure reduction by using SynergyFinder 3.0 and probability sum test in vivo

**DOI:** 10.1002/prp2.1064

**Published:** 2023-02-22

**Authors:** Tian Xia, Lu‐Lu Xu, Peng‐Yue Guo, Wan‐Ting Shi, Yan‐Qiong Cheng, Ai‐Jun Liu

**Affiliations:** ^1^ Department of Pharmacology, School of Pharmacy Naval Medical University Shanghai China; ^2^ Institute of Pharmacy Yueyang Hospital of Integrated Traditional Chinese and Western Medicine, Shanghai University of Traditional Chinese Medicine Shanghai China; ^3^ Department of Clinical Pharmacy Naval Medical University Shanghai China

**Keywords:** amlodipine, candesartan, hypertension, probability sum test, SynergyFinder 3.0, telmisartan

## Abstract

This study was designed to evaluate the synergism of two couples of antihypertensive drugs (amlodipine + telmisartan and amlodipine + candesartan) on blood pressure reduction in vivo by both SynergyFinder 3.0 and probability sum test. Spontaneously hypertensive rats were treated with intragastric administration of amlodipine (0.5, 1, 2, and 4 mg/kg), telmisartan (4, 8, and 16 mg/kg), candesartan (1, 2, and 4 mg/kg), nine combinations for amlodipine and telmisartan, and nine combinations for amlodipine and candesartan. The control rats were treated by 0.5% carboxymethylcellulose sodium. Blood pressure was recorded continuously up to 6 h after administration. Both SynergyFinder 3.0 and the probability sum test were used to evaluate the synergistic action. The synergisms calculated by SynergyFinder 3.0 are consistent with the probability sum test both in two different combinations. There is an obviously synergistic interaction between amlodipine and telmisartan or candesartan. The combinations of amlodipine and telmisartan (2 + 4 and 1 + 4 mg/kg) and amlodipine and candesartan (0.5 + 4 and 2 + 1 mg/kg) might exert an optimum synergism against hypertension. Compared with the probability sum test, SynergyFinder 3.0 is more stable and reliable to analyze the synergism.

## Graphical abstract

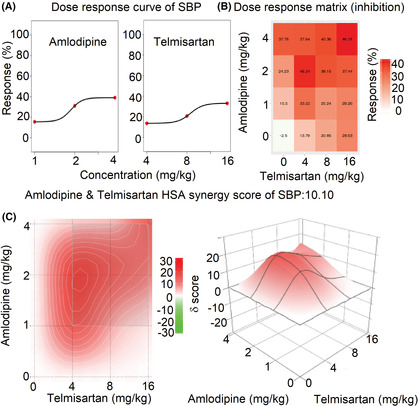

**Abbreviations**
BRAID: Bivariate Response to Additive Interacting Doses
CMC: carboxymethylcellulose sodium
DBP: diastolic blood pressure
MBP: mean blood pressure
MuSyC: Multidimensional Synergy of Combinations
SBP: systolic blood pressure
SD: standard deviation
SHRs: spontaneously hypertensive rats

## 1 INTRODUCTION

Drug combinations have become a standard therapy for many complex diseases, including cancers and cardiovascular diseases.^1^, ^2^ The combination with different mechanism drugs might produce synergistic interaction. It can enhance the treatment efficacy and avoid the acquisition of monotherapy resistance. Good combination therapy also reduces the dose of each drug. It is helpful to minimize the clinical and metabolic side effects of each individual component in larger dosage.^3^

SynergyFinder, a web‐application, was put forward in 2017 and has been widely used for the discovery of novel synergistic drug combinations.^4^, ^5^ SynergyFinder was designed to calculate the synergism between anti‐cancer medicines first. It also can be used in many precision medicine areas, targeted drug combination discovery.^6^ Recently, we reported that it can be used for anti‐platelet aggregation in vitro.^7^ It is unclear whether this method can be used to evaluate the synergism of antihypertensive drugs in vivo.

For the combination of different antihypertensive drugs, the probability sum test (*q* test) has been suggested to evaluate the synergism.^8^, ^9^ This test derives from classic probability analysis, and several groups have suggested that it may be useful for evaluating the synergism of combinations of two drugs in vivo.^8^, ^9^, ^10^, ^11^, ^12^, ^13^ However, there are some disadvantages. For example, about 20% data are discarded during the calculation. This elimination might affect the accuracy of the synergistic conclusion.

In the present study, both SynergyFinder 3.0 (the latest upgrade) and the probability sum test were performed to analyze the synergism on blood pressure reduction in vivo. Two couples of antihypertensive drugs (amlodipine + telmisartan and amlodipine + candesartan) were used in spontaneously hypertensive rats (SHRs). We mainly focused in the difference between the probability sum test and SynergyFinder 3.0 for the evaluation of synergism on blood pressure reduction.

## 2 MATERIALS AND METHODS

### 2.1 Animals

Male SHRs (aged 3 months) were purchased from Beijing Vital River Laboratory Animal Technology Co., Ltd. The rats were housed with controlled temperature (23–25°C) and lighting (8:00 AM to 8:00 PM light, 8:00 PM to 8:00 AM dark) and with free access to food and tap water. All the animals used in this experiment received humanitarian concern. All animal experiments were approved by the Committee on ethics of biomedicine of Naval Medical University and were conducted according to the National Institutes of Health Guidelines for the Care and Use of Laboratory Animals.

### 2.2 Blood pressure continuous recording in conscious rats

The operation and measurement were conducted as described previously in detail.^2^, ^14^ Briefly, rats were anesthetized by inhalation of 5% isoflurane and maintained with 2% isoflurane. The lower abdominal aorta was catheterized via the left femoral artery with a polyethylene catheter full of heparin (100 U/mL) for the recording of blood pressure. The catheters were tunneled subcutaneously, exposed through the interscapular skin and fixed. Then the animals were treated by meloxicam (2 mg/kg, subcutaneous injection) and penicillin (80 000 IU per animal, intramuscular injection) to relieve pain and prevent infection for 2 days. After 2 days recovery (with free access to food and tap water), rats were placed individually in cylindrical cages for blood pressure recording. The aortic catheter was connected to a pressure transducer via a swivel allowing rats to move freely. Beat‐to‐beat blood pressure signals and heart period were digitized by the MPA‐HBBS system (Alcott Biotech Co. Ltd) mounted in computer. After approximately 3 h habituation, the blood pressure and heart period signals were determined online. The data of blood pressure from 12:00 to 13:00 are used as the baseline (before administration). Then, at 13:00, the drug was given via the catheter of gastric fistula. The time reaching the maximum reduction in the blood pressure of each group was about half hour. Systolic blood pressure (SBP), diastolic blood pressure (DBP), and mean blood pressure (MBP) values from every heart beat were recorded from 0.5 h after administration up to 6 h.

*Experiment 1 protocol*: The effect of a single dose or different combinations of amlodipine and telmisartan on blood pressure reduction in SHRs.

Male SHRs were divided into 15 groups (*n* = 10 per group) and administered by the following drugs: amlodipine (1, 2 and 4 mg/kg), telmisartan (4, 8 and 16 mg/kg), and the combinations (1 + 4, 1 + 8, 1 + 16, 2 + 4, 2 + 8, 2 + 16, 4 + 4, 4 + 8 and 4 + 16 mg/kg).

The drugs were dissolved in the 0.5% carboxymethylcellulose sodium (CMC) and were given into the stomach through a catheter. The blood pressure was recorded according aforementioned method. The blood pressure values an hour before administration and mean values 6 h after administration were recorded to analyze.

*Experiment 2 protocol*: The effect of a single dose or different combinations of amlodipine and candesartan on blood pressure reduction in SHRs.

Male SHRs were divided into 13 groups (*n* = 10 per group) and administered by the following drugs: amlodipine (0.5 mg/kg), candesartan (1, 2 and 4 mg/kg) and the combinations (0.5 + 1, 0.5 + 2, 0.5 + 4, 1 + 1, 1 + 2, 1 + 4, 2 + 1, 2 + 2 and 2 + 4 mg/kg). The drugs were dissolved in the 0.5% CMC and were given into the stomach through a catheter. The blood pressure was recorded according aforementioned method. Blood pressure data of other 2 amlodipine alone groups (1 and 2 mg/kg) were got from experiment 1.

Another 10 SHRs were treated by vehicle (0.5% CMC) with the same volume as a control group. The blood pressure was recorded accordingly. The blood pressure values 1 h before administration and mean values 6 h after administration were recorded to analyze.

### 2.3 SynergyFinder 3.0

An excel was set up. There are several lines, including “Pair Index”, “Drug 1”, “Drug 2”, “dose 1”, “dose 2”, “Response” and “Dose Unit”. “Pair Index” represents how many pairs of drugs. “Drug 1” and “Drug 2” are the name of drugs in a dose‐response, while “dose 1” and “dose 2” are the doses of the drugs in combination. “Response” means the effect of drug combinations at the doses input by “dose 1” or “dose 2”. It is the reduction percentage of blood pressure. The range of the “Response” is expected from 0 to 100. The “Dose Unit” means the unit of drugs' dose.

The inhibition ratio of blood pressure reduction was used for SynergyFinder 3.0 system. Inhibition ratio = (blood pressure after administration − blood pressure before administration) × 3.33/blood pressure before administration × 100%. Unlike tumor cells, blood pressure cannot be reduced or inhibited by 100%. About 30% reduction in blood pressure is most appropriate in clinical.^15^ So we defined a 30% reduction in blood pressure as 100% inhibition. Dose‐effect curves for each drug on blood pressure were drawn and the half maximal inhibitory dose were calculated by using Prism software, version 8 (GraphPad). Choosing the percentage reduction as the phenotypic response, the blood pressure reduction of each drug combination was analyzed using SynergyFinder 3.0 (https://synergyfinder.fimm.fi).^5^

Briefly, the mean percentage of blood pressure reduction of a pair of antihypertensive drugs, and their combinations at different doses were input respectively. According to the software recommendation, highest single agent (HSA) reference model was selected in this study. This model states the expected combination effect is the maximum of the single drug responses at corresponding concentrations. Finally, the comprehensive synergy score and synergy maps of drug combination were obtained automatically by the software using HSA model. The synergy score was used to evaluate the efficacy of the combination. According to the user guide in the website, synergy score was evaluated as follows: scores <−10: the interaction between two drugs was likely to be antagonistic; from −10 to 10: the interaction between two drugs was likely to be additive; and > 10: the interaction between two drugs was likely to be synergistic.^7^

The synergy scoring can be visualized as either a two‐dimensional or a three‐dimensional interaction surface over the dose matrix. The depth of the color reveals the degree of percentage of blood pressure reduction in the two‐dimensional image and the height of the 3D drug interaction landscape is standardizing as the percentage of blood pressure reduction to show the comparison of the degrees of interaction among drug combinations.

### 2.4 Probability sum test

The probability sum test was used to evaluate the synergism of the combination according to the references.^10^, ^11^, ^12^ Compared with the control group, the experimental groups with a decrease in blood pressure >15 mmHg were defined as responders. Similarly, rats with a decrease in blood pressure <12 mmHg (80% of 15 mmHg) were defined as non‐responders and blood pressure data ranging from 12 to 15 mmHg were discarded. We calculate the *q* value as follows: *q* = *P*_A+B_/(*P*_A_ + *P*_B_ − *P*_A_ × *P*_B_). A and B represent drug A and drug B. *P* is the percentage of responders in each group. *P*_A+B_ is the responders' real percentage and (*P*_A_ + *P*_B_ − *P*_A_ × *P*_B_) is the expected response ratio. (*P*_A_ + *P*_B_) is the sum of the probabilities when drug A and drug B are used alone. *P*_A_ × *P*_B_ is the probability of ratios responding to both drugs when they were used alone. We considered that the combination was synergistic when *q* was >1.15 and the combination was antagonistic when *q* was <0.85. When *q* was between 0.85 and 1.15, the combination was additive.

### 2.5 Statistical analysis

The investigators were blinded to the animal groups when they assessed the blood pressure and analyzed the synergistic score using SynergyFinder 3.0 or the probability sum test. The animals were randomly assigned by using the random permutations table. Continuous variables were expressed as mean ± standard deviation (SD). Student's *t*‐test (two‐tailed paired *t*‐test) was used for comparisons between before and after drug administration. *p* < .05 was considered statistically significant.

## 3 RESULTS

### 3.1 Effects of amlodipine, telmisartan alone, and their combination on SBP, DBP, and MBP in SHRs

**FIGURE 1:**
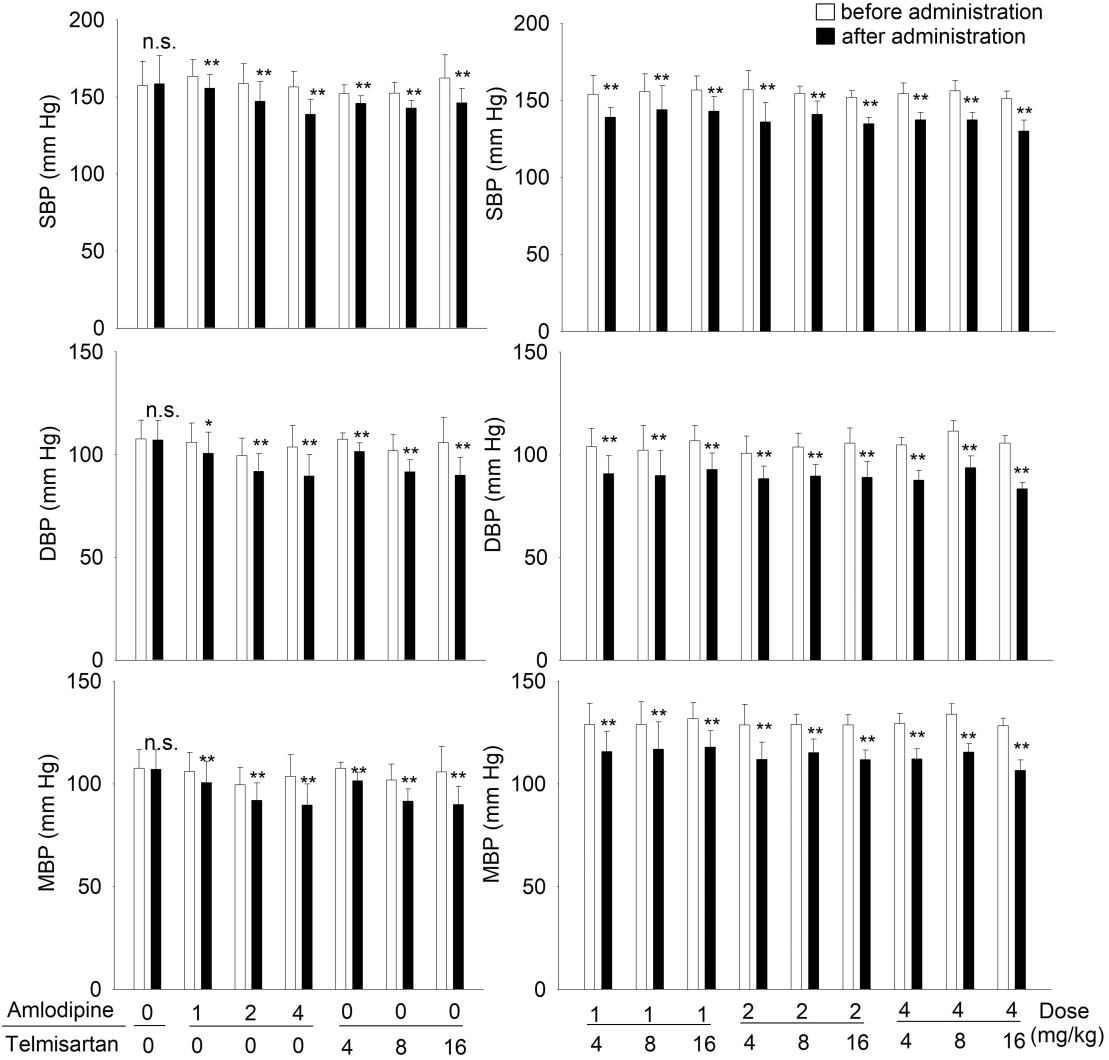
Effects of a single dose of amlodipine (1, 2 and 4 mg/kg) and telmisartan (4, 8 and 16 mg/kg) alone and their combination on the SBP, DBP and MBP in SHRs. Open bars mean before administration; solid bars mean after administration. Values are expressed as mean ± SD. *p < .05, **p < .01 versus before administration.

The effects of a single dose of amlodipine and telmisartan alone or in their combinations on SBP, DBP, and MBP are shown in Figure 1. In amlodipine alone treatment groups, compared to before administration, SBP values in 1, 2, and 4 mg/kg amlodipine groups were significantly reduced by approximately 8, 12, and 18 mmHg, respectively (Student's *t*‐test, *p* < .05). DBP values were significantly reduced by 5, 8, and 14 mmHg, respectively. MBP values were reduced by 6, 10, and 16 mmHg, respectively. In telmisartan treatment groups (4, 8, and 16 mg/kg), SBP values showed significant reduction by approximately 6, 10, and 16 mmHg, respectively. DBP was reduced by 16, 10, and 16 mmHg, and MBP was reduced by 6, 10, and 16 mmHg, respectively. Blood pressure values were also significantly reduced by the different combinations (Student's *t*‐test, *p* < .01).

In combination groups with amlodipine 1 mg/kg, adding telmisartan (4, 8, and 16 mg/kg) significantly reduced SBP by 15, 12, and 14 mmHg, respectively. In combination groups with amlodipine 2 mg/kg, adding telmisartan (4, 8, and 16 mg/kg) significantly reduced SBP by 21, 14, and 17 mmHg, respectively. In combination groups with amlodipine 4 mg/kg, adding telmisartan (4, 8, and 16 mg/kg) significantly reduced SBP by 17, 19, and 21 mmHg, respectively. The similar results were also got from DBP and MBP.

No significant change in blood pressure was observed in control rats compared with the blood pressure before administration.

### 3.2 Effects of amlodipine, candesartan alone, and their combination on SBP, DBP, and MBP in SHRs

**FIGURE 2:**
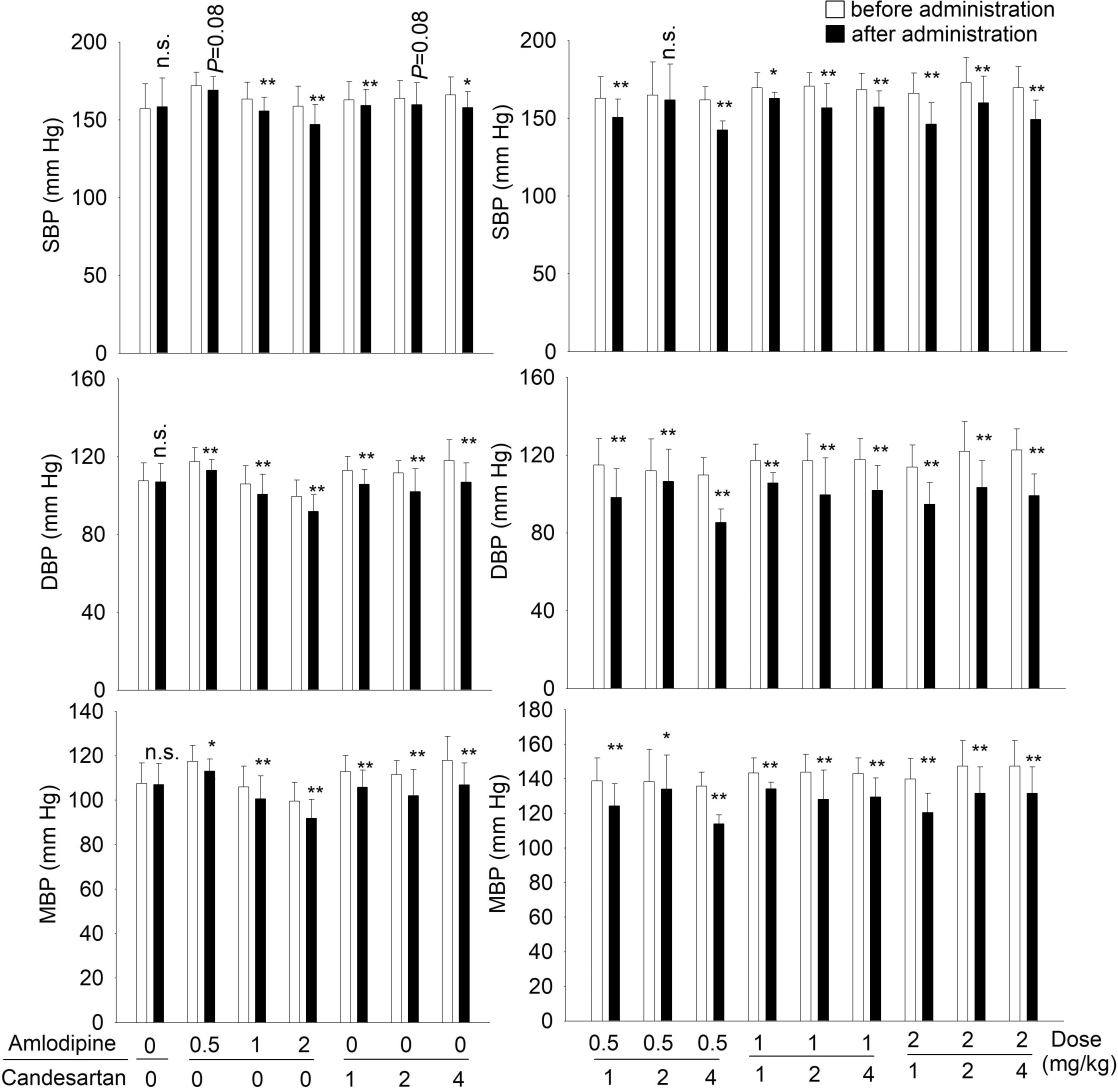
Effects of a single dose of amlodipine (0.5, 1 and 2 mg/kg) and candesartan (1, 2 and 4 mg/kg) alone and their combination on the SBP, DBP and MBP in SHRs. Open bars mean before administration; solid bars mean after administration. Values are expressed as mean ± SD. *p < .05, **p < .01 versus before administration.

The effects of amlodipine and candesartan alone or in their combinations on SBP, DBP, and MBP are shown in Figure 2. SBP values in 0.5 mg/kg amlodipine group were reduced by 3 mmHg (*p* = .08). DBP and MBP values were both reduced by 4 mmHg (Student's *t*‐test, *p* < .05). In candesartan treatment groups (1, 2, and 4 mg/kg), SBP values showed reduction by approximately 4, 4, and 8 mmHg, respectively. DBP was significantly reduced by 7, 10, and 11 mmHg, and MBP was significantly reduced by 5, 7, and 10 mmHg, respectively. Blood pressure values were significantly reduced by the different combinations (Student's *t*‐test, *p* < .05) except for one combination group (amlodipine 0.5 mg/kg + candesartan 2 mg/kg).

In combination groups with amlodipine 0.5 mg/kg, adding candesartan (1, 2, and 4 mg/kg) reduced SBP by 8, 3, and 9 mmHg, respectively. In combination groups with amlodipine 1 mg/kg, adding candesartan (1, 2, and 4 mg/kg) significantly reduced SBP by 7, 14, and 11 mmHg, respectively. In combination groups with amlodipine 2 mg/kg, adding candesartan (1, 2, and 4 mg/kg) significantly reduced SBP by 20, 13, and 20 mmHg, respectively. The significant reduction was also got from DBP and MBP.

### 3.3 The synergistic effects of amlodipine and telmisartan by SynergyFinder 3.0

**FIGURE 3:**
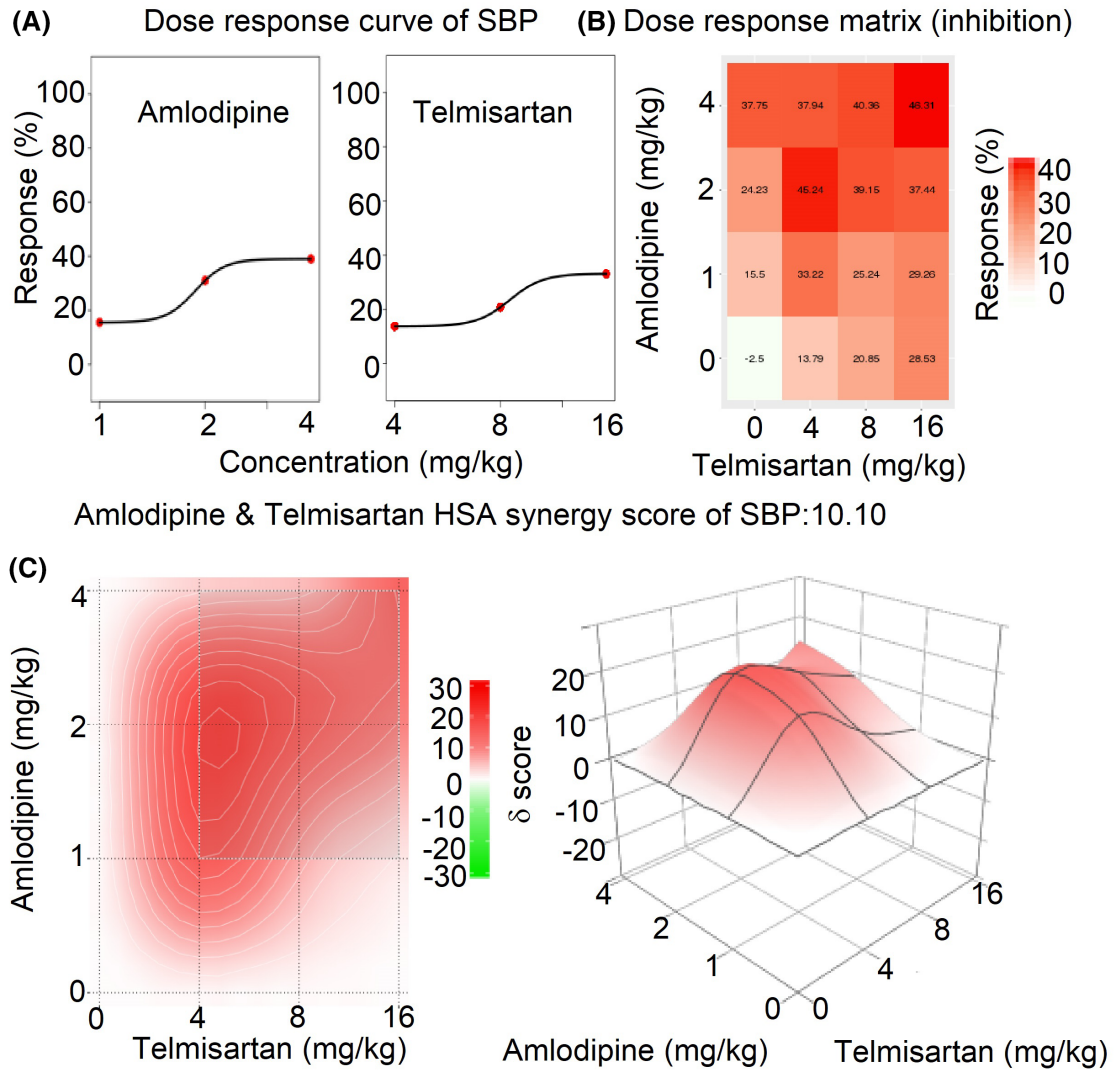
The drug interaction test for amlodipine and telmisartan on SBP by SynergyFinder 3.0. (A) the curve of the dose‐inhibition response for amlodipine & telmisartan output. *X*‐axis reflects the concentration of amlodipine & telmisartan, *Y*‐axis reflects the inhibition ratio on SBP. (B) dose‐response matrix (inhibition) for amlodipine & telmisartan. The inhibition ratio is positively relative to the degree of red. (C) the drug interaction landscapes based on the HSA model on SBP. The synergy score is showed by red (>0) and green (<0). Left is plane figure and right is solid figure.

**FIGURE 4:**
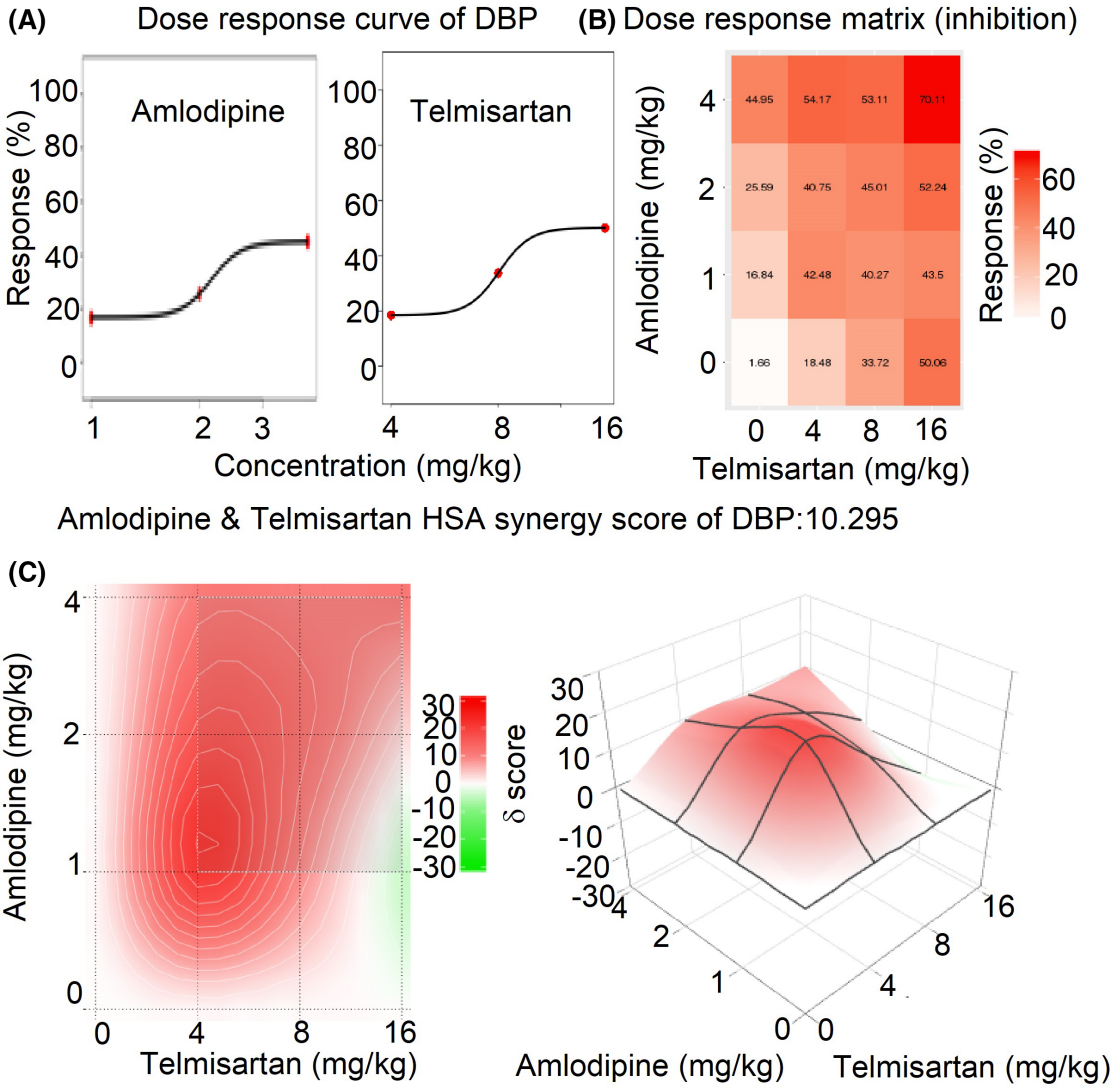
The drug interaction test for amlodipine and telmisartan on DBP by SynergyFinder 3.0. (A) the curve of the dose‐inhibition response for amlodipine & telmisartan output. *X*‐axis reflects the concentration of amlodipine & telmisartan, *Y*‐axis reflects the inhibition ratio on DBP. (B) dose‐response matrix (inhibition) for amlodipine & telmisartan. The inhibition ratio is positively relative to the degree of red. (C) the drug interaction landscapes based on the HSA model on DBP. The synergy score is showed by red (>0) and green (<0). Left is plane figure and right is solid figure.

**FIGURE 5:**
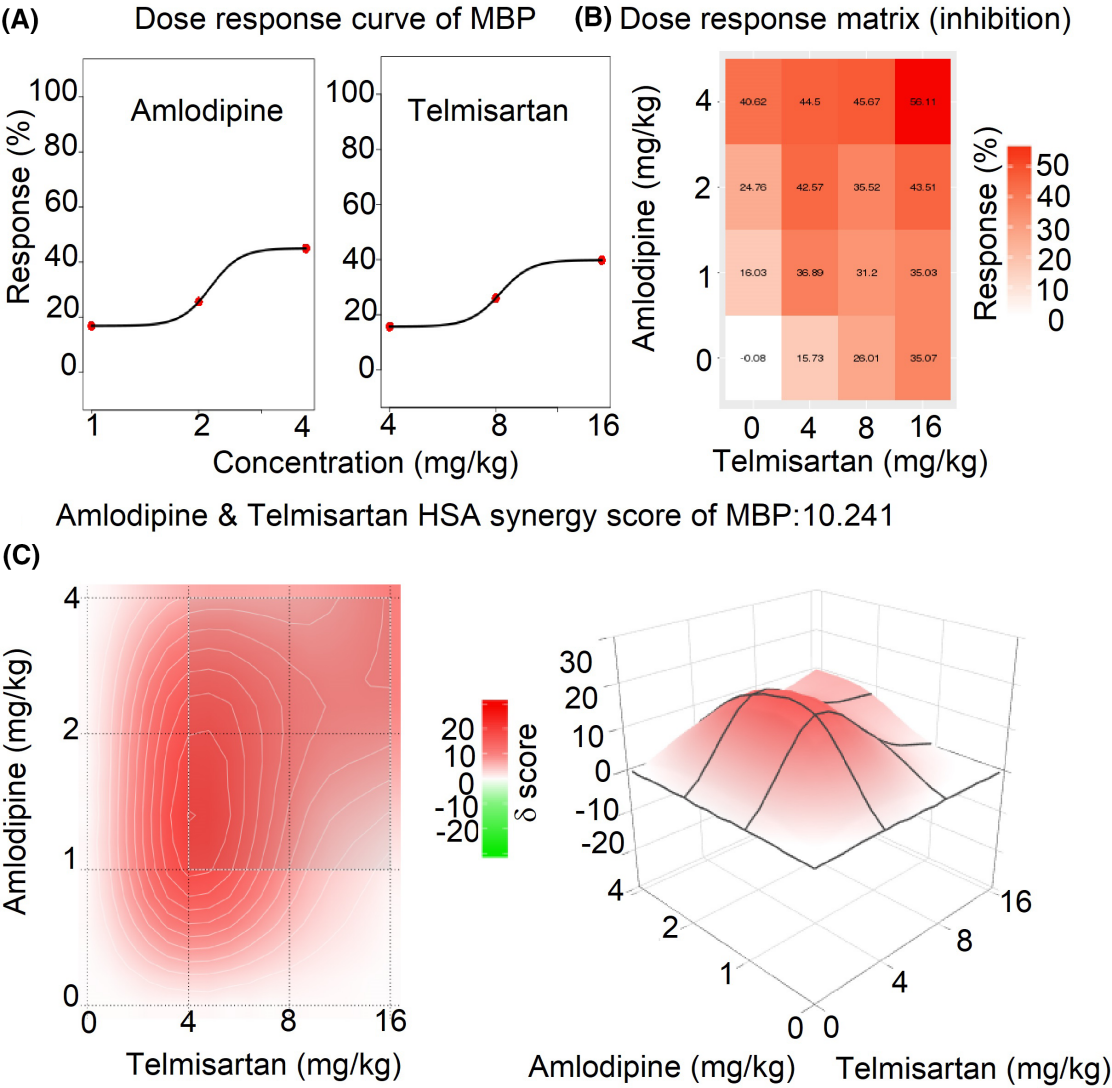
The drug interaction test for amlodipine and telmisartan on MBP by SynergyFinder 3.0. (A) the curve of the dose‐inhibition response for amlodipine & telmisartan output. *X*‐axis reflects the concentration of amlodipine & telmisartan, *Y*‐axis reflects the inhibition ratio on MBP. (B) dose‐response matrix (inhibition) for amlodipine & telmisartan. The inhibition ratio is positively relative to the degree of red. (C) the drug interaction landscapes based on the HSA model on MBP. The synergy score is showed by red (>0) and green (<0). Left is plane figure and right is solid figure.

The mean dose‐response of SBP, DBP, and MBP in rats administrated with amlodipine and telmisartan or their combinations are shown in Table S1–S3. The comprehensive synergy score and synergy maps of amlodipine and telmisartan combination are shown in Figures 3, 4, 5. The reduction ratio of blood pressure is positive relative to the dose of amlodipine or telmisartan (Figures 3, 4, 5). For SBP, DBP, and MBP, the comprehensive synergy scores of amlodipine combined with telmisartan are 10.10, 10.30, and 10.24, respectively, which indicates that amlodipine and telmisartan exhibit synergistic effect in these doses for blood pressure reduction. When the combined dose is 2 mg/kg amlodipine +4 mg/kg telmisartan, the synergy scores are 19.94, 16.70, and 17.93 for SBP, DBP, and MBP, respectively. The scores also reach a high level when the dose is 1 mg/kg amlodipine and 4 mg/kg telmisartan(15.96, 21.63, and 18.69 for SBP, DBP, and MBP, respectively). The data indicate that these two combinations (2 + 4 and 1 + 4 mg/kg) might exert an optimum synergistic effect.

### 3.4 The synergistic effects of amlodipine and candesartan by SynergyFinder 3.0

**FIGURE 6:**
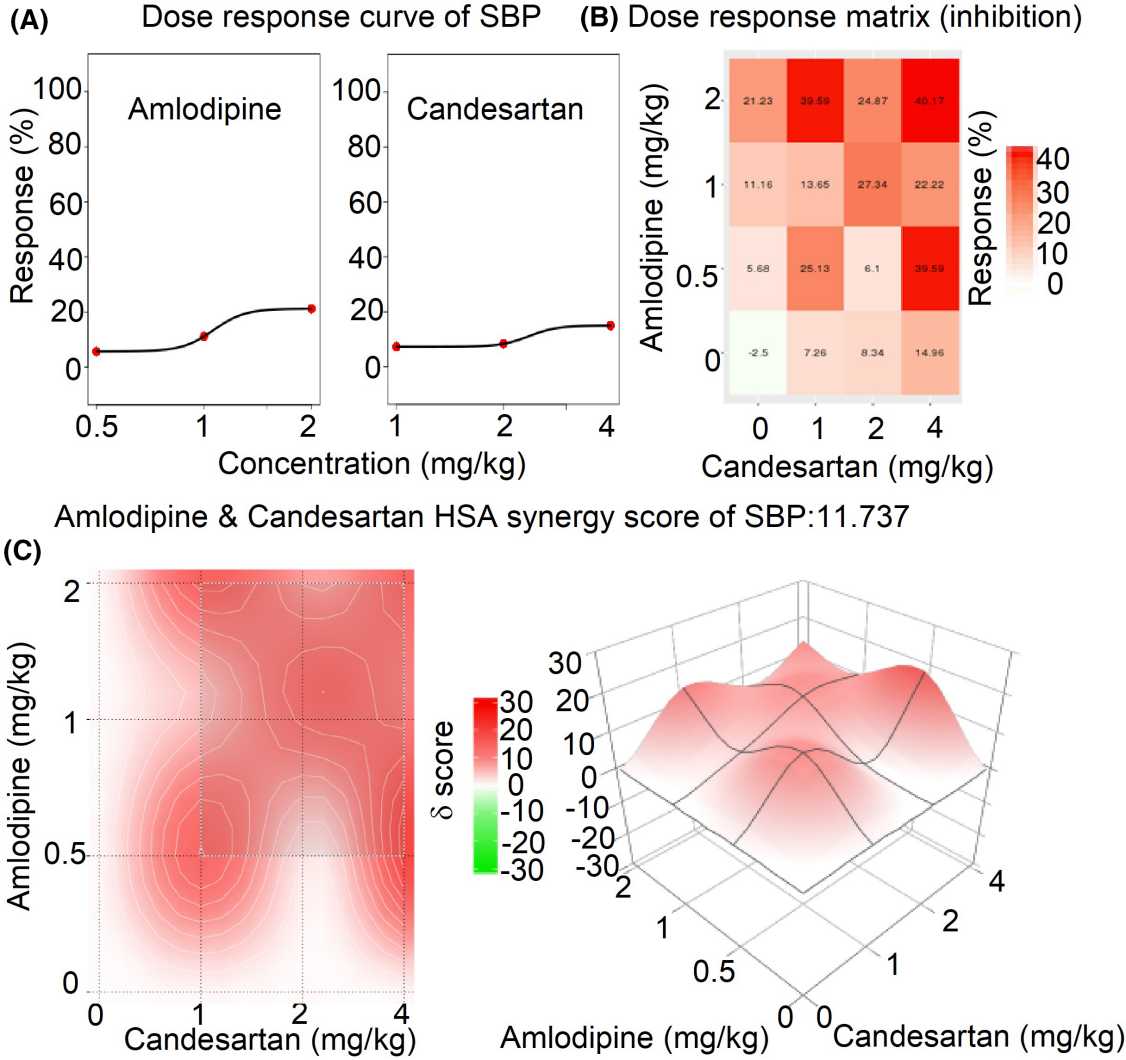
The drug interaction test for amlodipine and candesartan on SBP by SynergyFinder 3.0. (A) the curve of the dose‐inhibition response for amlodipine & candesartan output. *X*‐axis reflects the concentration of amlodipine & candesartan, *Y*‐axis reflects the inhibition ratio on SBP. (B) dose‐response matrix (inhibition) for amlodipine & candesartan. The inhibition ratio is positively relative to the degree of red. (C) The drug interaction landscapes based on the HSA model on SBP. The synergy score is showed by red (>0) and green (<0). Left is plane figure and right is solid figure.

**FIGURE 7:**
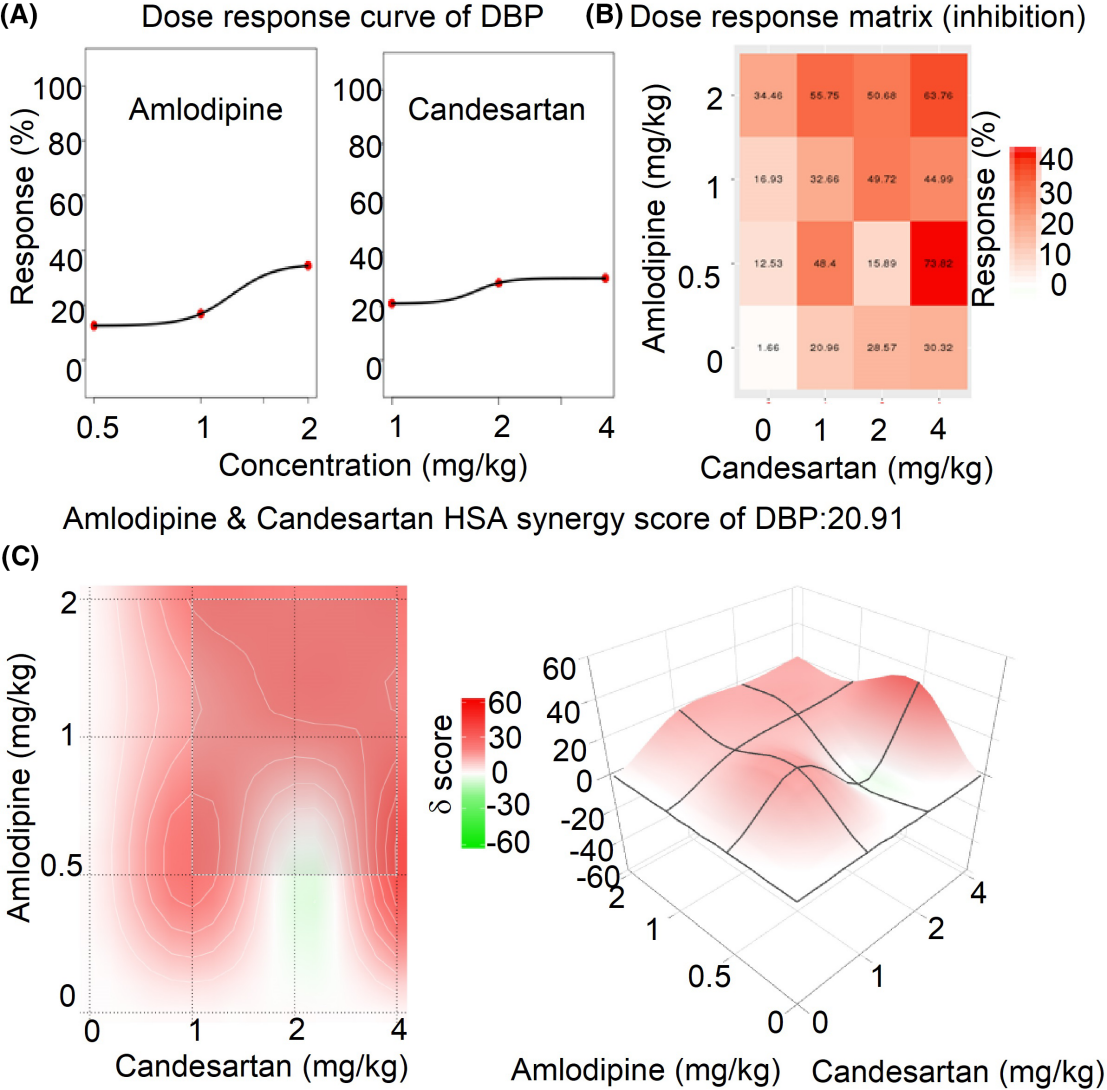
The drug interaction test for amlodipine and candesartan on DBP by SynergyFinder 3.0. (A) the curve of the dose‐inhibition response for amlodipine & candesartan output. *X*‐axis reflects the concentration of amlodipine & candesartan, *Y*‐axis reflects the inhibition ratio on DBP. (B) dose‐response matrix (inhibition) for amlodipine & candesartan. The inhibition ratio is positively relative to the degree of red. (C) the drug interaction landscapes based on the HSA model on DBP. The synergy score is showed by red (>0) and green (<0). Left is plane figure and right is solid figure.

**FIGURE 8:**
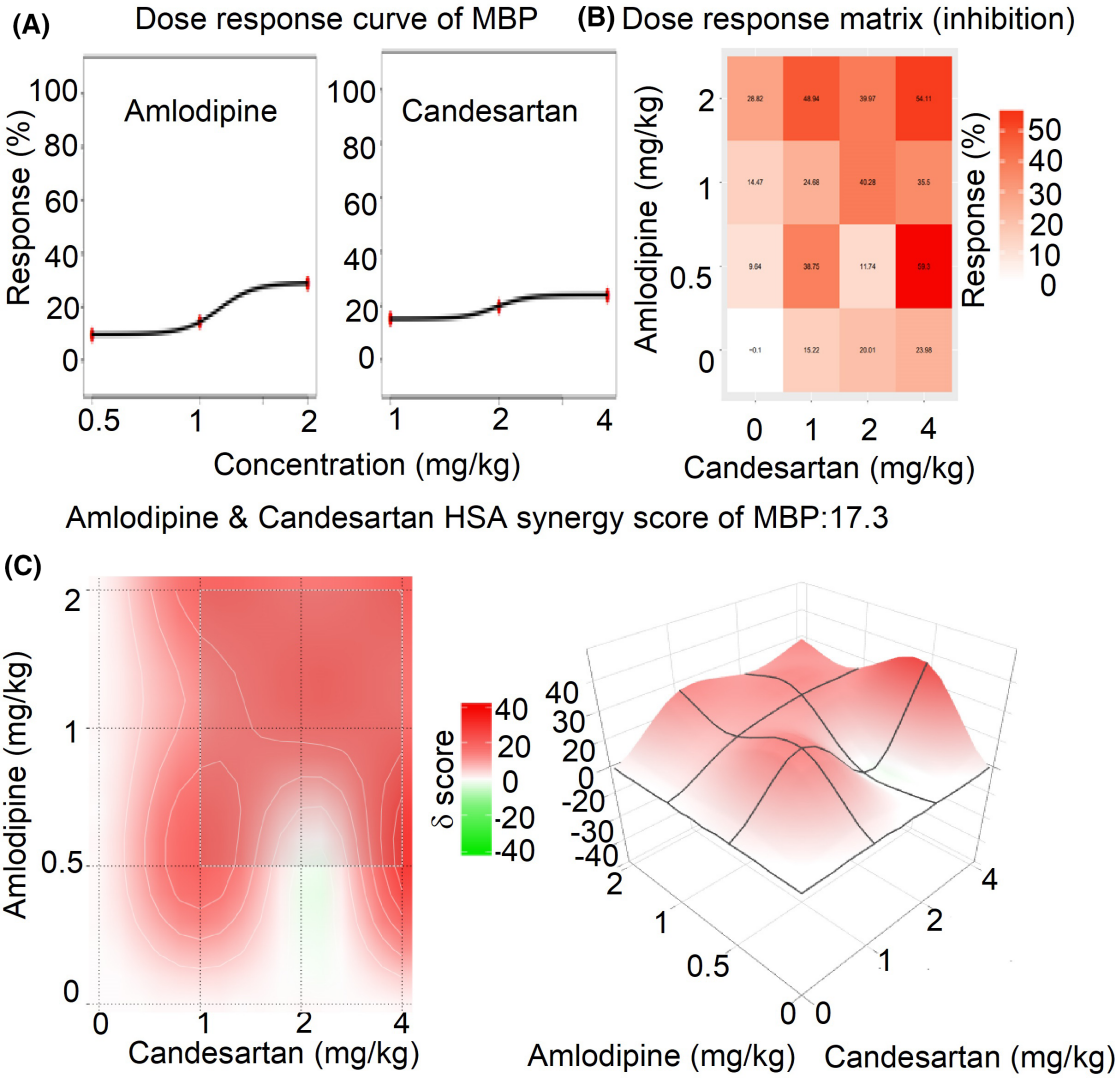
The drug interaction test for amlodipine and candesartan on MBP by SynergyFinder 3.0. (A) the curve of the dose‐inhibition response for amlodipine & candesartan output. *X*‐axis reflects the concentration of amlodipine & candesartan, *Y*‐axis reflects the inhibition ratio on MBP. (B) dose‐response matrix (inhibition) for amlodipine & candesartan. The inhibition ratio is positively relative to the degree of red. (C) the drug interaction landscapes based on the HSA model on MBP. The synergy score is showed by red (>0) and green (<0). Left is plane figure and right is solid figure.

The mean dose‐response of SBP, DBP, and MBP in rats administrated with amlodipine and candesartan or their combinations are shown in Table S4–S6. The comprehensive synergy score and synergy maps of amlodipine and candesartan combination are shown in Figures 6, 7, 8. The reduction ratio of blood pressure is positive relative to the dose of amlodipine or candesartan (Figures 6, 7, 8). For SBP, DBP, and MBP, the comprehensive synergy scores of amlodipine combined with candesartan are 11.74, 20.91, and 17.30, respectively, which indicates that amlodipine and telmisartan exhibit synergistic effect in these doses for blood pressure reduction. When the combined dose is 0.5 mg/kg amlodipine and 4 mg/kg candesartan, the synergy scores are 19.06, 35.55, and 28.11 for SBP, DBP, and MBP, respectively. These data indicate that the combination (0.5 + 4 mg/kg) might exert an optimum synergistic effect.

### 3.5 Synergistic effects of amlodipine and telmisartan by probability sum test

Table 1 presents the results of the probability sum test of SBP, DBP, and MBP in data from SHRs treated with amlodipine, telmisartan, and their combinations. For SBP, the *q* values only in two combination groups (amlodipine + telmisartan, 1 + 8 and 1 + 16 mg/kg) are <1.15 (1.05 and 0.90). These data indicate that all the other combination groups might have a synergistic effect on SBP. For DBP, the *q* values only in one group (1 + 8 mg/kg) are <1.15 (0.97). All *q* values of MBP are larger than 1.15. In the combination group (2 + 4 mg/kg), the *q* values (6 for SBP and 6.25 for MBP) are larger than any other groups. These data indicate that this combination (2 + 4 mg/kg) might exert an optimum synergistic effect.

**TABLE 1:** The probability sum test in rats administrated with amlodipine and telmisartan or their combinations.

Amlodipine (mg/kg)	Telmisartan (mg/kg)	*P*_SBP_	*P*_DBP_	*P*_MBP_	*q* SBP	*q* DBP	*q* MBP
0	4	0%	0%	0%			
0	8	22.2%	33.3%	25.0%			
0	16	42.9%	37.5%	42.8%			
1	0	22.2%	11.1%	0%			
2	0	12.5%	0%	10%			
4	0	60.0%	57.1%	66.7%			
1	4	66.7%	57.1%	57.1%	3.00	5.15	/
1	8	28.6%	42.9%	37.5%	1.05	0.97	1.50
1	16	50.0%	66.7%	71.4%	0.90	1.50	1.67
2	4	75.0%	44.4%	62.5%	6.00	/	6.25
2	8	71.4%	71.4%	66.7%	2.24	2.15	2.05
2	16	100%	100%	100%	2.00	2.67	2.06
4	4	77.8%	87.5%	87.5%	1.30	1.53	1.31
4	8	100%	100%	100%	1.45	1.40	1.33
4	16	100%	100%	100%	1.30	1.37	1.24

*Note*: *P*_SBP_, *P*_DBP_, and *P*_MBP_ were the percentages of the effective decrease (≥15 mmHg) in SBP, DBP and MBP respectively by amlodipine, telmisartan and their combinations. The values of *q* > 1.15 represents synergistic; *q* < 0.85 represents antagonistic.

### 3.6 Synergistic effects of amlodipine and candesartan by probability sum test

Table 2 shows the probability sum test of SBP, DBP, and MBP in data from SHRs treated with amlodipine and candesartan. For SBP, the *q* values only in two combination groups (amlodipine + candesartan, 1 + 1 and 1 + 2 mg/kg) are <1.15 (1.13 and 1.11). All the other *q* values of combination groups are larger than 1.15. These groups might have a synergistic effect on SBP. For DBP and MBP, the *q* values only in one group (0.5 + 2 mg/kg) are <1.15 (0.50 and 1.00 for DBP and MBP, respectively). All the other *q* values are larger than 1.15. For SBP and MBP, the *q* values in two combination groups (2 + 1 and 0.5 + 4 mg/kg) are larger than any other group. All these data indicates that these two combinations (2 + 1 and 0.5 + 4 mg/kg) might exert an optimum synergistic effect.

**TABLE 2:** The probability sum test in rats administrated with amlodipine and candesartan or their combinations.

Amlodipine (mg/kg)	Candesartan (mg/kg)	*P*_SBP_	*P*_DBP_	*P*_MBP_	*q* SBP	*q* DBP	*q* MBP
0	1	0%	0%	0%			
0	2	10.0%	22.2%	11.1%			
0	4	12.5%	37.5%	14.3%			
0.5	0	0%	0%	0%			
1	0	22.2%	11.1%	0%			
2	0	12.5%	0%	10.0%			
0.5	1	50.0%	60.0%	60.0%	/	/	/
0.5	2	0%	11.1%	11.1%	/	0.50	1.00
0.5	4	77.8%	100%	100%	6.22	2.67	7.00
1	1	25.0%	37.5%	33.3%	1.13	3.38	/
1	2	33.3%	44.4%	40.0%	1.11	1.44	3.60
1	4	37.5%	70.0%	62.5%	1.17	1.58	4.38
2	1	87.5%	100%	88.9%	7.00	/	8.89
2	2	44.4%	62.5%	55.6%	2.09	2.81	2.78
2	4	80.0%	80.0%	80.0%	3.41	2.13	3.50

*Note*: *P*_SBP_, *P*_DBP_, and *P*_MBP_ were the percentages of the effective decrease (≥15 mmHg) in SBP, DBP and MBP respectively by amlodipine, candesartan and their combinations. The values of *q* > 1.15 represents synergistic; *q* < 0.85 represents antagonistic.

## 4 DISCUSSION

Amlodipine is a calcium channel blocker (CCB).^16^ Telmisartan and candesartan belong to angiotensin II type 1 (AT (1)) receptor blocker (ARB).^17^ The action mechanisms between amlodipine and telmisartan or candesartan are also quite different. They might have synergistic effect on blood pressure reduction.

It's difficult to determine the synergistic interaction in practice for antihypertensive drugs, especially in vivo. For anti‐cancer drugs, there are some new methods to evaluate the synergism of drugs combination.^18^, ^19^, ^20^, ^21^ For example, the Combenefit is a new software tool. It uses classical Synergy models and enables the visualization and analysis of drug combination effects in cancer cells model.^18^ DeepSynergy is an accurate tool for synergy analysis. DeepSynergy learns to distinguish different cancer cell lines and find certain drugs combinations. The results will be output in visual two‐dimensional graph.^19^ Multidimensional Synergy of Combinations (MuSyC) is a formalism based on a generalized, multi‐dimensional Hill‐equation. It will decouple synergistic potency and efficacy. MuSyC is also mainly used in calculating the synergy between anti‐cancer medicines. The results can be presented in coordinate system.^20^ The Bivariate Response to Additive Interacting Doses (BRAID) model is designed to maintain a balance between versatility and simplicity for anti‐cancer medicines. Through eight parameter model in cancer cells, BRAID quantifies the synergy and makes it visual.^21^ More evidence is needed to clarify whether these methods can be used for in vivo research in other diseases, including hypertension.

SynergyFinder 3.0 might be an answer. SynergyFinder was designed to calculate the synergism between anti‐cancer medicines firstly. It has been widely used for the discovery of novel synergistic drug combinations and updated to version 3.0.^4^, ^5^ It's designed to enable unbiased identification of synergistic combinations from high‐throughput experimental data. It shows the highest synergy among all the combinations and searches further development and testing toward safe and effective treatment options. It also can be used in many precision medicine areas, targeted drug combination discovery.^6^ Recently, we reported it can be used for anti‐platelet aggregation in vitro.^7^

In this study, we selected two different couples of antihypertensive drugs (amlodipine plus telmisartan or amlodipine plus candesartan) as an example, and calculated the synergistic effect on blood pressure reduction by SynergyFinder 3.0 in vivo. The inhibition ratio of blood pressure reduction was used for SynergyFinder 3.0 system. For hypertensive patients, a 30% reduction in blood pressure is most appropriate in clinical. So we defined a 30% reduction in blood pressure as 100% inhibition. The results output by SynergyFinder 3.0 are more credible. The results are consistent with the probability sum test both in the combination of amlodipine and telmisartan or amlodipine and candesartan (effective reduction of blood pressure defined as ≥15 mmHg). In the combination groups of amlodipine and telmisartan, the *q* values in two doses groups (2 + 4 and 1 + 4 mg/kg) are larger than any other group. The synergy scores by SynergyFinder 3.0 are also highest in these two groups. The two combinations groups of amlodipine and candesartan (0.5 + 4 and 2 + 1 mg/kg) also get the similar results.

The probability sum test also has been widely used to analyze the synergism of the combinations of two antihypertensive drugs.^8^, ^9^, ^10^, ^11^, ^12^, ^13^ The synergistic or antagonistic effect is mainly defined by the *q* value (*P*_A+B_/(*P*_A_ + *P*_B_ − *P*_A_ × *P*_B_)). However, there are some disadvantages for this method compared with SynergyFinder 3.0. First, the definition of responders for blood pressure reduction. We usually define a decrease in blood pressure >15 or 20 mmHg as responders.^2^, ^10^ *P* is the percentage of responders in each group. When the reduction percentage is equal to zero, it will have a great impact on the *q* values, which might cause a big difference between different responders. Second, the elimination of some data might affect the accuracy of *q* values. In this experiment, a reduction in blood pressure >15 mmHg is defined as responders. A decrease <12 mmHg (80% of 15 mmHg) is defined as non‐responders and data ranging from 12 to 15 mmHg are discarded, which might affect the synergistic conclusion.

In SynergyFinder 3.0, all the data are input to the system and are reflected in synergy pictures and scores. SynergyFinder 3.0 combines multiple synergy scoring models and obtains a novel consensus synergy score. During the data processing, the false positive results will be removed with an improved outlier detection functionality.^5^ Hence, individual extreme data will not have much influences on synergy scores. Different with the probability sum test, the synergy scores of SBP, DBP, and MBP by SynergyFinder 3.0 are high consistent.

In conclusion, SynergyFinder 3.0 is more stable and reliable to analyze the synergism on blood pressure reduction in vivo than the probability sum test. There is an obviously synergistic interaction between amlodipine & telmisartan or candesartan. The combinations of amlodipine & telmisartan (2 + 4 and 1 + 4 mg/kg) and amlodipine & candesartan (0.5 + 4 and 2 + 1 mg/kg) might exert an optimum synergistic effect against hypertension.

## AUTHOR CONTRIBUTIONS

Conceived the idea: Ai‐Jun Liu. Designed the experiments: Ai‐Jun Liu and Yan‐Qiong Cheng. Performed the experiments: Tian Xia, Lu‐Lu Xu, and Wan‐Ting Shi. Analyzed the data: Tian Xia, Lu‐Lu Xu, Yan‐Qiong Cheng, and Ai‐Jun Liu. Wrote the paper: Ai‐Jun Liu and Tian Xia.

## CONFLICT OF INTEREST STATEMENT

The authors declare that they have no known competing financial interests or personal relationships that could have appeared to influence the work reported in this paper.

## ETHICS STATEMENT

All experiments were approved by the Committee on ethics of biomedicine of Naval Medical University and were conducted according to the National Institutes of Health Guidelines for the Care and Use of Laboratory Animals.

## Supporting information


Table S1–S6

## Data Availability

Available per request.
