# Supplementary material for: Synergism of amlodipine and telmisartan or candesartan on blood pressure reduction by using SynergyFinder 3.0 and probability sum test in vivo
Source: Pharmacol Res Perspect. 2023 Feb 22;11(2):e01064. doi: 10.1002/prp2.1064 (PMC9944853; doi:10.1002/prp2.1064)
Supplement: Supplementary file 1 — Table S1–S6 [file PRP2-11-e01064-s001.docx]

**Supplementary table 1** The mean dose-response of SBP in rats administrated with amlodipine and telmisartan or their combinations.

| **PairIndex** | **Drug1** | **Drug2** | **Conc1** | **Conc2** | **Response (%)** | **ConcUnit** |
| --- | --- | --- | --- | --- | --- | --- |
| 1 | Amlodipine | Telmisartan | 0 | 0 | -2.50 | mg/kg |
| 1 | Amlodipine | Telmisartan | 0 | 4 | 13.79 | mg/kg |
| 1 | Amlodipine | Telmisartan | 0 | 8 | 20.85 | mg/kg |
| 1 | Amlodipine | Telmisartan | 0 | 16 | 28.53 | mg/kg |
| 1 | Amlodipine | Telmisartan | 1 | 0 | 15.50 | mg/kg |
| 1 | Amlodipine | Telmisartan | 1 | 4 | 33.22 | mg/kg |
| 1 | Amlodipine | Telmisartan | 1 | 8 | 25.24 | mg/kg |
| 1 | Amlodipine | Telmisartan | 1 | 16 | 29.26 | mg/kg |
| 1 | Amlodipine | Telmisartan | 2 | 0 | 24.23 | mg/kg |
| 1 | Amlodipine | Telmisartan | 2 | 4 | 45.24 | mg/kg |
| 1 | Amlodipine | Telmisartan | 2 | 8 | 39.15 | mg/kg |
| 1 | Amlodipine | Telmisartan | 2 | 16 | 37.44 | mg/kg |
| 1 | Amlodipine | Telmisartan | 4 | 0 | 37.75 | mg/kg |
| 1 | Amlodipine | Telmisartan | 4 | 4 | 37.94 | mg/kg |
| 1 | Amlodipine | Telmisartan | 4 | 8 | 40.36 | mg/kg |
| 1 | Amlodipine | Telmisartan | 4 | 16 | 46.31 | mg/kg |

Conc: concentration. Response is the mean inhibition ration (%). Inhibition ratio = (blood pressure after administration - blood pressure before administration) × 3.33 / blood pressure before administration × 100%.

**Supplementary table 2** The mean dose-response of DBP in rats administrated with amlodipine and telmisartan or their combinations.

| **PairIndex** | **Drug1** | **Drug2** | **Conc1** | **Conc2** | **Response (%)** | **ConcUnit** |
| --- | --- | --- | --- | --- | --- | --- |
| 1 | Amlodipine | Telmisartan | 0 | 0 | 1.66 | mg/kg |
| 1 | Amlodipine | Telmisartan | 0 | 4 | 18.48 | mg/kg |
| 1 | Amlodipine | Telmisartan | 0 | 8 | 33.72 | mg/kg |
| 1 | Amlodipine | Telmisartan | 0 | 16 | 50.06 | mg/kg |
| 1 | Amlodipine | Telmisartan | 1 | 0 | 16.84 | mg/kg |
| 1 | Amlodipine | Telmisartan | 1 | 4 | 42.48 | mg/kg |
| 1 | Amlodipine | Telmisartan | 1 | 8 | 40.27 | mg/kg |
| 1 | Amlodipine | Telmisartan | 1 | 16 | 43.50 | mg/kg |
| 1 | Amlodipine | Telmisartan | 2 | 0 | 25.59 | mg/kg |
| 1 | Amlodipine | Telmisartan | 2 | 4 | 40.75 | mg/kg |
| 1 | Amlodipine | Telmisartan | 2 | 8 | 45.01 | mg/kg |
| 1 | Amlodipine | Telmisartan | 2 | 16 | 52.24 | mg/kg |
| 1 | Amlodipine | Telmisartan | 4 | 0 | 44.95 | mg/kg |
| 1 | Amlodipine | Telmisartan | 4 | 4 | 54.17 | mg/kg |
| 1 | Amlodipine | Telmisartan | 4 | 8 | 53.11 | mg/kg |
| 1 | Amlodipine | Telmisartan | 4 | 16 | 70.11 | mg/kg |

Conc: concentration. Response is the mean inhibition ration (%). Inhibition ratio = (blood pressure after administration - blood pressure before administration) × 3.33 / blood pressure before administration × 100%.

**Supplementary table 3** The mean dose-response of MBP in rats administrated with amlodipine and telmisartan or their combinations.

| **PairIndex** | **Drug1** | **Drug2** | **Conc1** | **Conc2** | **Response (%)** | **ConcUnit** |
| --- | --- | --- | --- | --- | --- | --- |
| 1 | Amlodipine | Telmisartan | 0 | 0 | -0.08 | mg/kg |
| 1 | Amlodipine | Telmisartan | 0 | 4 | 15.73 | mg/kg |
| 1 | Amlodipine | Telmisartan | 0 | 8 | 26.01 | mg/kg |
| 1 | Amlodipine | Telmisartan | 0 | 16 | 35.07 | mg/kg |
| 1 | Amlodipine | Telmisartan | 1 | 0 | 16.03 | mg/kg |
| 1 | Amlodipine | Telmisartan | 1 | 4 | 36.89 | mg/kg |
| 1 | Amlodipine | Telmisartan | 1 | 8 | 31.20 | mg/kg |
| 1 | Amlodipine | Telmisartan | 1 | 16 | 35.03 | mg/kg |
| 1 | Amlodipine | Telmisartan | 2 | 0 | 24.76 | mg/kg |
| 1 | Amlodipine | Telmisartan | 2 | 4 | 42.57 | mg/kg |
| 1 | Amlodipine | Telmisartan | 2 | 8 | 35.52 | mg/kg |
| 1 | Amlodipine | Telmisartan | 2 | 16 | 43.51 | mg/kg |
| 1 | Amlodipine | Telmisartan | 4 | 0 | 40.62 | mg/kg |
| 1 | Amlodipine | Telmisartan | 4 | 4 | 44.50 | mg/kg |
| 1 | Amlodipine | Telmisartan | 4 | 8 | 45.67 | mg/kg |
| 1 | Amlodipine | Telmisartan | 4 | 16 | 56.11 | mg/kg |

Conc: concentration. Response is the mean inhibition ration (%). Inhibition ratio = (blood pressure after administration - blood pressure before administration) × 3.33 / blood pressure before administration × 100%.

**Supplementary table 4** The mean dose-response of SBP in rats administrated with amlodipine and candesartan or their combinations.

| **PairIndex** | **Drug1** | **Drug2** | **Conc1** | **Conc2** | **Response (%)** | **ConcUnit** |
| --- | --- | --- | --- | --- | --- | --- |
| 1 | Amlodipine | Candesartan | 0 | 0 | -2.50 | mg/kg |
| 1 | Amlodipine | Candesartan | 0 | 1 | 7.26 | mg/kg |
| 1 | Amlodipine | Candesartan | 0 | 2 | 8.34 | mg/kg |
| 1 | Amlodipine | Candesartan | 0 | 4 | 14.96 | mg/kg |
| 1 | Amlodipine | Candesartan | 0.5 | 0 | 5.68 | mg/kg |
| 1 | Amlodipine | Candesartan | 0.5 | 1 | 25.13 | mg/kg |
| 1 | Amlodipine | Candesartan | 0.5 | 2 | 6.10 | mg/kg |
| 1 | Amlodipine | Candesartan | 0.5 | 4 | 39.59 | mg/kg |
| 1 | Amlodipine | Candesartan | 1 | 0 | 11.16 | mg/kg |
| 1 | Amlodipine | Candesartan | 1 | 1 | 13.65 | mg/kg |
| 1 | Amlodipine | Candesartan | 1 | 2 | 27.34 | mg/kg |
| 1 | Amlodipine | Candesartan | 1 | 4 | 22.22 | mg/kg |
| 1 | Amlodipine | Candesartan | 2 | 0 | 21.23 | mg/kg |
| 1 | Amlodipine | Candesartan | 2 | 1 | 39.59 | mg/kg |
| 1 | Amlodipine | Candesartan | 2 | 2 | 24.87 | mg/kg |
| 1 | Amlodipine | Candesartan | 2 | 4 | 40.17 | mg/kg |

Conc: concentration. Response is the mean inhibition ration (%). Inhibition ratio = (blood pressure after administration - blood pressure before administration) × 3.33 / blood pressure before administration × 100%.

**Supplementary table 5** The mean dose-response of DBP in rats administrated with amlodipine and candesartan or their combinations.

| **PairIndex** | **Drug1** | **Drug2** | **Conc1** | **Conc2** | **Response (%)** | **ConcUnit** |
| --- | --- | --- | --- | --- | --- | --- |
| 1 | Amlodipine | Candesartan | 0 | 0 | 1.66 | mg/kg |
| 1 | Amlodipine | Candesartan | 0 | 1 | 20.96 | mg/kg |
| 1 | Amlodipine | Candesartan | 0 | 2 | 28.57 | mg/kg |
| 1 | Amlodipine | Candesartan | 0 | 4 | 30.32 | mg/kg |
| 1 | Amlodipine | Candesartan | 0.5 | 0 | 12.53 | mg/kg |
| 1 | Amlodipine | Candesartan | 0.5 | 1 | 48.40 | mg/kg |
| 1 | Amlodipine | Candesartan | 0.5 | 2 | 15.89 | mg/kg |
| 1 | Amlodipine | Candesartan | 0.5 | 4 | 73.82 | mg/kg |
| 1 | Amlodipine | Candesartan | 1 | 0 | 16.93 | mg/kg |
| 1 | Amlodipine | Candesartan | 1 | 1 | 32.66 | mg/kg |
| 1 | Amlodipine | Candesartan | 1 | 2 | 49.72 | mg/kg |
| 1 | Amlodipine | Candesartan | 1 | 4 | 44.99 | mg/kg |
| 1 | Amlodipine | Candesartan | 2 | 0 | 34.46 | mg/kg |
| 1 | Amlodipine | Candesartan | 2 | 1 | 55.75 | mg/kg |
| 1 | Amlodipine | Candesartan | 2 | 2 | 50.68 | mg/kg |
| 1 | Amlodipine | Candesartan | 2 | 4 | 63.76 | mg/kg |

Conc: concentration. Response is the mean inhibition ration (%). Inhibition ratio = (blood pressure after administration - blood pressure before administration) × 3.33 / blood pressure before administration × 100%.

**Supplementary table 6** The mean dose-response of MBP in rats administrated with amlodipine and candesartan or their combinations.

| **PairIndex** | **Drug1** | **Drug2** | **Conc1** | **Conc2** | **Response (%)** | **ConcUnit** |
| --- | --- | --- | --- | --- | --- | --- |
| 1 | Amlodipine | Candesartan | 0 | 0 | -0.10 | mg/kg |
| 1 | Amlodipine | Candesartan | 0 | 1 | 15.22 | mg/kg |
| 1 | Amlodipine | Candesartan | 0 | 2 | 20.01 | mg/kg |
| 1 | Amlodipine | Candesartan | 0 | 4 | 23.98 | mg/kg |
| 1 | Amlodipine | Candesartan | 0.5 | 0 | 9.64 | mg/kg |
| 1 | Amlodipine | Candesartan | 0.5 | 1 | 38.75 | mg/kg |
| 1 | Amlodipine | Candesartan | 0.5 | 2 | 11.74 | mg/kg |
| 1 | Amlodipine | Candesartan | 0.5 | 4 | 59.30 | mg/kg |
| 1 | Amlodipine | Candesartan | 1 | 0 | 14.47 | mg/kg |
| 1 | Amlodipine | Candesartan | 1 | 1 | 24.68 | mg/kg |
| 1 | Amlodipine | Candesartan | 1 | 2 | 40.28 | mg/kg |
| 1 | Amlodipine | Candesartan | 1 | 4 | 35.50 | mg/kg |
| 1 | Amlodipine | Candesartan | 2 | 0 | 28.82 | mg/kg |
| 1 | Amlodipine | Candesartan | 2 | 1 | 48.94 | mg/kg |
| 1 | Amlodipine | Candesartan | 2 | 2 | 39.97 | mg/kg |
| 1 | Amlodipine | Candesartan | 2 | 4 | 54.11 | mg/kg |

Conc: concentration. Response is the mean inhibition ration (%). Inhibition ratio = (blood pressure after administration - blood pressure before administration) × 3.33 / blood pressure before administration × 100%.
